# Short-Circuited Turn Fault Diagnosis in Transformers by Using Vibration Signals, Statistical Time Features, and Support Vector Machines on FPGA

**DOI:** 10.3390/s21113598

**Published:** 2021-05-21

**Authors:** Jose R. Huerta-Rosales, David Granados-Lieberman, Arturo Garcia-Perez, David Camarena-Martinez, Juan P. Amezquita-Sanchez, Martin Valtierra-Rodriguez

**Affiliations:** 1 ENAP-Research Group, CA-Sistemas Dinámicos y Control, Laboratorio de Sistemas y Equipos Eléctricos (LaSEE), Facultad de Ingeniería, Universidad Autónoma de Querétaro (UAQ), Campus San Juan del Río, Río Moctezuma 249, Col. San Cayetano, San Juan del Río, CP 76807, Mexico; roberto.huerta@enap-rg.org (J.R.H.-R.); juan.amezquita@enap-rg.org (J.P.A.-S.); 2 ENAP-Research Group, CA-Fuentes Alternas y Calidad de la Energía Eléctrica, Departamento de Ingeniería Electromecánica, Tecnológico Nacional de México, Instituto Tecnológico Superior de Irapuato (ITESI), Carr. Irapuato-Silao km 12.5, Colonia El Copal, Irapuato, Guanajuato, CP 36821, Mexico; david.granados@enap-rg.org; 3 ENAP-Research Group, División de Ingeniería, Universidad de Guanajuato, Campus Irapuato-Salamanca, Carretera Salamanca-Valle de Santiago km 3.5 + 1.8 km, Comunidad de Palo Blanco, Salamanca, Guanajuato, CP 36885, Mexico; arturo.garcia@enap-rg.org (A.G.-P.); david.camarena@enap-rg.org (D.C.-M.)

**Keywords:** fault diagnosis, support vector machine, linear discriminant analysis, FPGA, short-circuit fault, transformer, vibration signals

## Abstract

One of the most critical devices in an electrical system is the transformer. It is continuously under different electrical and mechanical stresses that can produce failures in its components and other electrical network devices. The short-circuited turns (SCTs) are a common winding failure. This type of fault has been widely studied in literature employing the vibration signals produced in the transformer. Although promising results have been obtained, it is not a trivial task if different severity levels and a common high-level noise are considered. This paper presents a methodology based on statistical time features (STFs) and support vector machines (SVM) to diagnose a transformer under several SCTs conditions. As STFs, 19 indicators from the transformer vibration signals are computed; then, the most discriminant features are selected using the Fisher score analysis, and the linear discriminant analysis is used for dimension reduction. Finally, a support vector machine classifier is employed to carry out the diagnosis in an automatic way. Once the methodology has been developed, it is implemented on a field-programmable gate array (FPGA) to provide a system-on-a-chip solution. A modified transformer capable of emulating different SCTs severities is employed to validate and test the methodology and its FPGA implementation. Results demonstrate the effectiveness of the proposal for diagnosing the transformer condition as an accuracy of 96.82% is obtained.

## 1. Introduction

In an electrical network, the transformer is one of the most valuable pieces of equipment. Although the transformer is a robust machine, it is under different electrical and mechanical stresses for inherent operating conditions. As well as under different weather conditions, which can produce different types of failures [1] and, consequently interrupt the continuity of the power supply and will influence the stability and safety of power systems [2]. The most common fault occurs in windings since they are among the most vulnerable internal components in a transformer [3,4]. Besides, the high electrodynamic forces that appear during short-circuited turns (SCTs) provoke winding deformations and core failures [5,6]. Hence, the development and successful application of monitoring systems and protection schemes for reducing the negative effects that the transformer faults produce on service and users represent a crucial task [7]. There are different techniques to diagnose a transformer, for instance: dissolved gas analysis (DGA), insulating quality, power factor testing, thermography, frequency response analysis (FRA), among others [4]. Although promising results have been obtained with these traditional methods, the vibration analysis (VA) method has received special interest in recent years because it represents an effective way to assess different applications as deterioration induction motors [8], bearing faults [9], civil structures [10], etc. In a transformer, principally because the vibration signals directly correlate with winding mechanical changes [11].

Several examples of VA techniques in literature can be separated into signal-based, model-based, and knowledge-based [12]. The signal-based algorithms extract information from vibration signals. The Fourier transform (FT) can extract information, which is a common tool for a frequency domain analysis. Bartoletti et al. [13] use FT to differentiate between a healthy transformer and an anomalous transformer. Meanwhile, Bagheri et al. [14] analyze the vibration spectra to detect short-circuit initiation. Even though the FT has presented good results, its performance decreases when the signal presents non-stationary events as monitored in the transformers [15]. In this regard, advanced signal processing has been introduced, specifically time-frequency domain techniques. Borucki [16] develops an assessment method for the mechanical structure of the core and windings transformer using the short-time Fourier Transform.

Meanwhile, Liu et al. [17] propose a methodology based on the wavelet transform (WT). Zhao and Xu [18] present a variation of WT. They use the empirical wavelet transform (EWT) to construct a time-frequency representation using the Hilbert transform. Finally, Wu et al. [19] present a time-scale-frequency analysis technique based on wavelet packet transform and Hilbert Huang transform. The major drawback to adopting WT-based approaches is the dependence on the adequate selection of the mother wavelet and decomposition level [20] because they change in each different application. On the other hand, model-based algorithms establish a direct relationship between the vibration and the input parameters. A model based on transformer tank vibration is developed by Garcia et al. [21]. Their analysis can detect winding deformation by measuring tank vibrations; besides, they obtain the most suitable place to acquire vibration signals from the transformer tank. Zheng et al. [11] present a model to diagnose mechanical faults in windings. The faults diagnosed are the winding clamping force and winding deformation. Other authors have investigated the winding deformation. For instance, Zhou et al. [22] develop a winding vibration model coupled with electromagnetic force. Their model distinguishes between faulty and normal windings. The major drawback of this approach is the incapability to diagnose in a transient state. On the other hand, works such as [23,24,25,26] have used knowledge-based algorithms to diagnose transformers, which are focused on pattern recognition and decision making. Hong et al. [23] present a probability-based classification model, which extracts two features from vibration signals, then a support vector machine (SVM) for classifying the transformer condition is used. However, the use of two features is sufficiently accurate to distinguish between some classes. Unfortunately, it is not suitable for applications where several fault levels are considered because the features are not always capable of maximizing the separation among classes. In [24], a new methodology for mapping an artificial neural network (ANN) into a rule-based fuzzy inference system (FIS) is proposed, i.e., they extract the rules for the FIS from a trained ANN. Another approach based on fractal dimension and data mining is presented in [25]. This method employs different fractal dimension algorithms as fault indicators and compares different classifiers. The best results are obtained by the K-nearest neighbor-based classifier, showing the capability to detect different fault severities of SCTs. In the work presented by Bigdeli et al. [26], an intelligent winding fault classification through transfer function analysis is developed. Their review of intelligent methods found that the SVM classifier is known as one of the best methods for solving classification problems. They compare the performance of SVM and ANN classifiers and conclude that SVM has better performance than ANN. They highlighted that the most important factor required for a successful SVM-based fault recognition is the proper selection of input features. 

The abovementioned works employ different features to carry out the diagnosis. For instance, in [26] they use mathematical indices from the frequency domain (i.e., frequency and amplitude variations), whereas in [23] they use features in both time and frequency domains (i.e., energy and frequency complexity). The work presented in [7] focuses on energies and entropies, obtaining good results in different transformer operations, i.e., in transient or steady states. On the other hand, the fractals indices presented in [25] demonstrated that can be an effective tool for the diagnosis of transformers. On the other hand, many other works have been focused on the use of statistical time features (STFs) and the proper selection of such input features. Consequently, efficient results have been obtained on applications related to rotatory machinery [27,28,29], sensor fault detection [30], civil structures [10], solar flare prediction [31], cardiac diseases [32], epileptic seizure detection [33], amputees limb motion [34], and neurodegenerative diseases [35], among others. In general, STFs are used to find and describe relevant signal properties and, at the same time, differentiate between different types of signals or classes, e.g., a healthy condition class and a fault condition class. Another advantage of the STFs is that they can be applied to non-stationary time signals [36], which are very common in real scenarios. The most common STFs are mean, standard deviation, variance, root mean square (RMS), skewness, and kurtosis [27,37]; in addition to them, other STFs such as square root mean (SRM), shape factor, log energy entropy (LEE), skewness factor, kurtosis factor, among others are prospective features to be considered in the diagnosis of different transformer winding conditions. Therefore, all of them will be explored to develop a tool based on certain STFs, which allows the proper analysis of vibration signals and, consequently, the diagnosis of SCTs conditions. On the other hand, it is worth noting that it is necessary to select appropriate features to maximize relevance and minimize redundancy into the available information to obtain a better performance, knowing that the construction of models from datasets with many features is more computationally demanding [38]. There are two processes to reduce the number of features: feature extraction and feature selection. For feature extraction, the classical methods include linear discriminant analysis (LDA), principal component analysis (PCA), and multidimensional scaling (MDS) [39]. On the other hand, a widely used feature reduction method is the Fisher score, which removes redundant features [40,41].

All the above-reviewed research works, and in this article, a methodology based on STFs, SVM, and its implementation on a field-programmable gate array (FPGA) to diagnose a transformer under different SCTs conditions using vibration signals is presented. The methodology emphasizes the feature selection to obtain the best features that properly describe the winding condition. These features should maximize the separation among classes. The study was carried out with real vibration signals acquired from a modified transformer with diverse conditions of SCTs. The range of SCTs is from 0 to 35 turns with steps of 5 turns, where the condition of 0 SCTs represents the healthy condition. The first step is to calculate the nineteen STFs from vibration signals; then, the set of features are examined by a feature set reduction approach to take the most representative subset of features. The reduction process consists of a feature selection by using the Fisher score analysis and the feature extraction by using the LDA. For these results, a classification algorithm based on SVM is proposed, which is compared with another common classification approach, i.e., ANN. Besides, the methodology implementation into an FPGA is presented to provide a system-on-a-chip solution for the future development of online monitoring systems. The selection of this technology is due to its parallelism, high speed, reconfigurability, and low cost [42], which make it a promising tool for the development of smart sensors [43]. The obtained results show the effectiveness of the proposal.

## 2. Theoretical Background

In this section, a brief description of the algorithms used in the proposed work is presented.

### 2.1. Transformer Vibration

The transformer vibrations are generated principally into the core and windings. The magnetostriction phenomenon produces the core vibrations, which change the shape of a ferromagnetic material under a magnetic field. The forces are induced into the core when the voltage is applied; it appears in a perpendicular direction to the core and occurs twice per cycle; hence, the fundamental frequency of core vibration is double of the transformer excitation voltage frequency [44]. The magnitude of the transformer core vibration ($F_{core}$) is proportional to the square of the excitation voltage (*V*) as seen in (1). Due to the non-linearity of magnetostriction, harmonics of high frequencies are induced with random magnitudes [45].

(1) $$F_{core}\propto V^{2}$$

On the other hand, winding vibration is caused by electromagnetic forces resulting from the interaction between the winding current and the magnetic leakage flux. These forces have components in axial and radial directions, which are proportional to the square of the current signal; consequently, the vibration acceleration ($F_{winding}$) is proportional to the square of the current (*I*) as shown in (2) [22]. Similar to the core, the main acceleration frequency is twice the fundamental frequency of the current. Also, some harmonics of the fundamental frequency source can appear due to the magnetizing current or some residual currents. Finally, the influence of other factors in transformer vibration has a relatively minor impact, e.g., the power factor exhibits a very small variation over time, and the temperature factor has very small fluctuations [46].

(2) $$F_{winding}\propto I^{2}$$

As can be seen, the vibration signals are directly related to the transformer performance; therefore, the application of STFs to characterize the properties of the vibration signal can help to determine the transformer condition.

### 2.2. Statistical Time Features

STFs extract information from a signal about the behavior of the system, i.e., a system with different operating conditions has signals with different statistical parameters. In this regard, the objective of feature extraction in fault detection is to obtain parameters that can correctly reflect the working condition of a system and, consequently, identify its fault patterns [36]. It is worth noting that these features have been satisfactorily applied to condition monitoring due to their simplicity, low computational burden, and, principally, the capability to estimate general trends [10,40].

In vibration signal analysis, some STFs such as mean, RMS, standard deviation, and variance to study differences between signals associated with a fault condition have been studied [36]; meanwhile, in not purely stationary signals, advanced statistical features such as skewness and kurtosis have been used to examine them, by measuring the deviation of a distribution from the normal distribution [47]. Despite obtaining promising results, many other features have to be investigated. Hence, in this work, the most representative STFs such as mean, maximum value, RMS, SRM, standard deviation, variance, shape factor with RMS and SRM, crest, latitude and impulse factor, skewness, skewness factor, kurtosis, kurtosis factor, normalized fifth and sixth moment, Shannon entropy, LEE (i.e., log energy entropy), which have been presented in different fields of research, are calculated and analyzed to properly describe the operating condition of the transformer with SCTs conditions. In Table 1, the mathematical formulation for the above-mentioned nineteen STFs are presented [29,39,48,49,50,51], where $x_{i}$ is the time signal for i=1,2…,N, and *N* is the number of data points. 

Although it is good to obtain as much information as possible about a system, the information provided by the STFs can be redundant to carry out a diagnostic; in this regard, the most relevant features for this particular goal have to be selected in a posterior processing step [52], as mentioned in next subsection.

### 2.3. Feature Reduction

The performance of a fault identification system depends on the input features. A small set of features does not ensure the complete description of the system, e.g., different transformer SCTs conditions, and they may drive misclassification. Meanwhile, many features may increase the capability of discrimination but do not ensure the addition of relevant information related to malfunctions, i.e., the information can be redundant. In addition, the construction of models from data sets with many features is more computationally demandant [53]. Hence, algorithms to discover significant information from features, avoid redundancy, and reduce the data sets must be applied. The feature reduction is performed through the process of feature extraction and feature selection [38].

#### 2.3.1. Feature Selection

Feature selection aims to maximize relevance and minimize redundancy into the information, taking a small feature subset from the original feature set [41]. In general, it is considered an approach to setoff filtering. The most used filter approaches are based on information, distance, consistency, and statistical measures, among others [49]. An effective strategy that has been successfully employed for different applications because of its good results and easiness of computation is the Fisher score analysis [41,49,54,55]. The Fisher score (FS) finds a subset of features with the maximum distance between classes while the distances between data points in the same class are as small as possible. The ${\mathrm{FS}}^{j}$ of the *j* feature is computed as [54]:

(22) $${\mathrm{FS}}^{j}=\frac{\sum_{i=1}^{c}n_{i}{\left(\mu_{i}^{j}-\mu^{j}\right)}^{2}}{\sum_{i=1}^{c}n_{i}{\left(\sigma_{i}^{j}\right)}^{2}}$$

where $\mu_{i}^{j}$, $\sigma_{i}^{j}$ and $n_{i}$ are the mean, standard deviation, and size of *i*th *c*-class of the *j*th feature, respectively, $\mu^{j}$ is the mean of the whole data set of the *j*th feature. After computing the FS, the top-ranked features have been selected and extracted.

#### 2.3.2. Feature Extraction

The feature extraction procedure transforms the original features into a new set constructed by the combinations of the original set (dimension reduction). Its purpose is to discover meaningful information in the new set. The most commonly used methods are the PCA (i.e., principal component analysis), [37,50,52], and the LDA (i.e., linear discriminant analysis) [56,57,58,59].

LDA is a supervised feature extraction technique that consists of a linear combination of the features, reducing the feature space, and scaling the features according to their importance. Besides, it deals with multi-class problems, has an easy implementation, and a clear physical interpretation. In general, the objective of LDA is to find a new lower-dimension projection to maximize the distance of samples from different classes and minimize the distance of samples from the same class.

The optimal projection vector **w** to obtain a well-separated class in a low-dimensional space must satisfy the separability maximization criterion among classes. It is given by the Fisher criterion [58]:

(23) $$w=\arg\max\left|\frac{w^{T}S_{b}w}{w^{T}S_{w}w}\right|$$

where $S_{b}$ is the between-class scatter matrix, which evaluates the separability of different classes, while $S_{w}$ is the within-class scatter matrix that evaluates the compactness within each class and the between-class scatter matrix. They are defined as [56]:

(24) $$S_{b}=\sum_{i=1}^{c}n_{i}\left(\mu_{i}-\mu \right){\left(\mu_{i}-\mu \right)}^{T}$$

(25) $$S_{w}=\sum_{i=1}^{c}\sum_{j=1}^{n_{i}}\left(x_{j}^{i}-\mu_{i}\right){\left(x_{j}^{i}-\mu_{i}\right)}^{T}$$

where $n_{i}$ is the number of samples in the *j*th *c*-class, $\mu_{i}=\frac{1}{n_{i}}\sum_{j=1}^{n_{i}}x_{j}^{i}$ denotes the mean feature of samples of the *i*th *c*-class, and $\mu =\frac{1}{n}\sum_{i=1}^{c}\sum_{j=1}^{i}x_{j}^{i}$ is the mean feature of all samples.

Solving Equation (23), the optimal projection vector **w** is the eigenvector corresponding to the maximum eigenvalue λ of [57]:

(26) $$S_{w}w=\lambda S_{b}w$$

For multi-class classification, the projection matrix **W** is constructed as a set of eigenvectors corresponding to the largest *k* eigenvalues $\left\{\lambda_{i}|i=1,2,\ldots ,k\right\}$. Finally, the feature extracted **Y** into a new low-dimensional space by the projection of the original data set **x** can be obtained as follows [40]:

(27) $$Y=W^{T}x$$

This new set of features contains the best information, improving the accuracy of the classification method [38]. 

### 2.4. Support Vector Machine

The application of SVMs in fault diagnosis has attracted the attention of researchers around the world due to their excellent classification performance on small samples, high dimension, and nonlinear problems. The SVM can solve linear classification problems easily, and when the classification problem is nonlinear, the SVM turns the nonlinear and inseparable classification problem into a nonlinear and separable classification problem [60].

The SVM is a linear classifier whose objective is to find an optimal hyperplane between two different classes to obtain a decision function to classify the samples in a specific class. The decision function is constructed as [30,61]

(28) $$g(x)=\omega^{T}x+\omega_{0}=0$$

where ω is the vector of weights, **x** is the input vector, and $\omega_{0}$ is the bias. The goal is to search the direction that gives the maximum possible margin between the support vectors and the hyperplane. The equations for the support vectors of each class are given as

(29) $$\begin{array}{l} \omega^{T}x+\omega_{0}\geq 1{,}^{}\forall x\in \omega_{1}\\ \omega^{T}x+\omega_{0}\leq -1,\forall x\in \omega_{2} \end{array}$$

where $\omega_{1}$ and $\omega_{2}$ correspond to each class. Then, to find the optimal hyperplane (training), the quadratic problem minimizing presented in Equation (30) has to be solved:

(30) $$J(\omega )\equiv \frac{1}{2}{\left\|\omega \right\|}^{2}$$

subject to

(31) $$y_{i}\left(\omega^{T}x_{i}+\omega_{0}\right)\geq 1_{,}$$

then, the final decision function can be obtained as

(32) $$\omega =\sum_{i=1}^{N}y_{i}\alpha_{i}x_{i},$$

where $\alpha_{i}$ are the Lagrange multipliers, $y_{i}=\pm 1$ is a class indicator for each training data $x_{i}$, which also are known as support vectors. In contrast, the optimal hyperplane classifier is known as a support vector machine. When nonlinear and non-separable patterns appear, the resulting linear classifier is

(33) $$g(x)={\sum_{i=1}^{N}y_{i}\alpha_{i}K\left(x_{i},x\right)+\omega_{0}}_{,}$$

where $x_{i}$ is assigned to $\omega_{1}$ if g(x)>1 or $\omega_{2}$ if g(x)<0, and $K\left(x_{i},x\right)$ is a kernel function. The most used kernel function is the radial basis function (RBF) [2,60]; therefore, it is used in this study, and it is defined as

(34) $$K\left(x_{i},x\right)=\exp{\left(-\frac{{\left\|x-x_{i}\right\|}^{2}}{\sigma^{2}}\right)}_{.}$$

**Figure 1:**
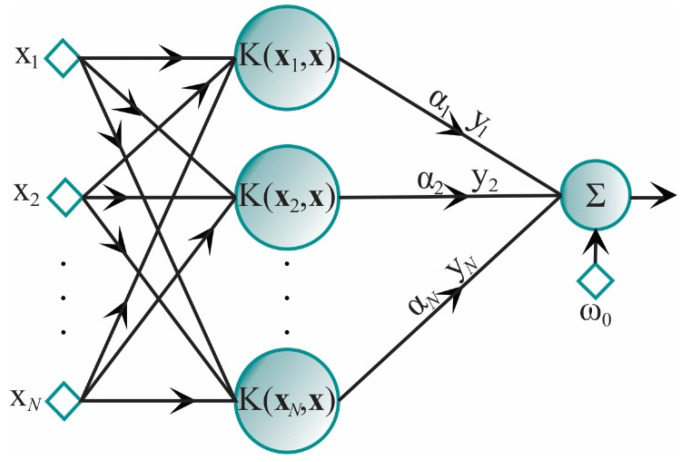
SVM architecture.

The parameter σ is called the kernel scale. To control the SVM generalization capability, a misclassification parameter *C* is also defined. This parameter controls the trade-off between the margin and the size of the slack variables. The selection of SVM parameters *C* and σ has an important influence on the classification accuracy. Both parameters are chosen by the user [26]. In Figure 1, the corresponding architecture of an SVM is shown. 

**Figure 2:**
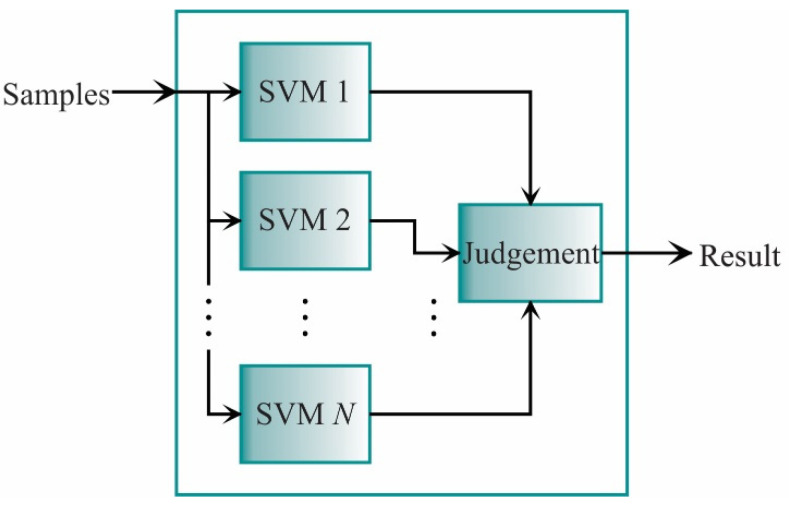
Scheme for the parallel SVM approach.

The SVM is commonly employed for binary classification; yet, it can also be used for multiple-class classification. For this last task, the multiple one-against-other classifiers are adopted. There are two approaches to carry out the multiple one-against-others classifications. The first approach is to put each classifier in series, whereas, in the second, the one-against-others SVMs are putting in parallel. In this work, the one-against-all approach is employed in a parallel way because the input sample can be evaluated by each classifier at the same time, saving processing time and exploiting the FPGA capabilities. The scheme for this approach is shown in Figure 2 [62].

Each classifier in Figure 2 is designed to separate one class from the rest. As aforementioned, it is expected that all points from the class $\omega_{i}$ in the ${\mathrm{SVM}}_{i}$ classifier yields g(x)>0, while a negative value is obtained for the rest of the classes. Then, **x** data is classified in $\omega_{i}$ if satisfy:

(35) $$\omega_{i}{}^{T}x+\omega_{i0}>\omega_{j}{}^{T}x+\omega_{j0,}\forall i\neq j$$

To evaluate the performance of the SVM, the *k*-fold cross-validation is utilized. The experiments are separated into *k* groups. The *i*th group is left out, and the others *k*-1 groups are used to train the SVM. The group left out is used to carry out the validation process and measure the error. The process is repeated *k* times with the (*i*-1)th validation data; where i=1,…,k. Finally, the error of each *k* validation is averaged [33]. In this work, five-fold cross-validation is used because it allows one to train the classifier with a large number of samples by class (80%), maintaining the error estimation and its variance in low values [63].

## 3. Proposed Methodology

**Figure 3:**
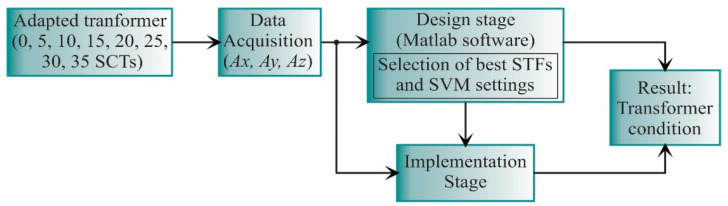
General block diagram for the proposed work.

The proposed work is carried out as shown in Figure 3. In general, the vibration signals from the transformer under different SCT fault conditions are acquired. Then, these signals are analyzed during the design stage to obtain the best STFs and the SVM settings, which will be implemented into the FPGA for hardware computation. The result of both the design and implementation stages is the transformer diagnosis. 

### 3.1. Design Stage

**Figure 4:**
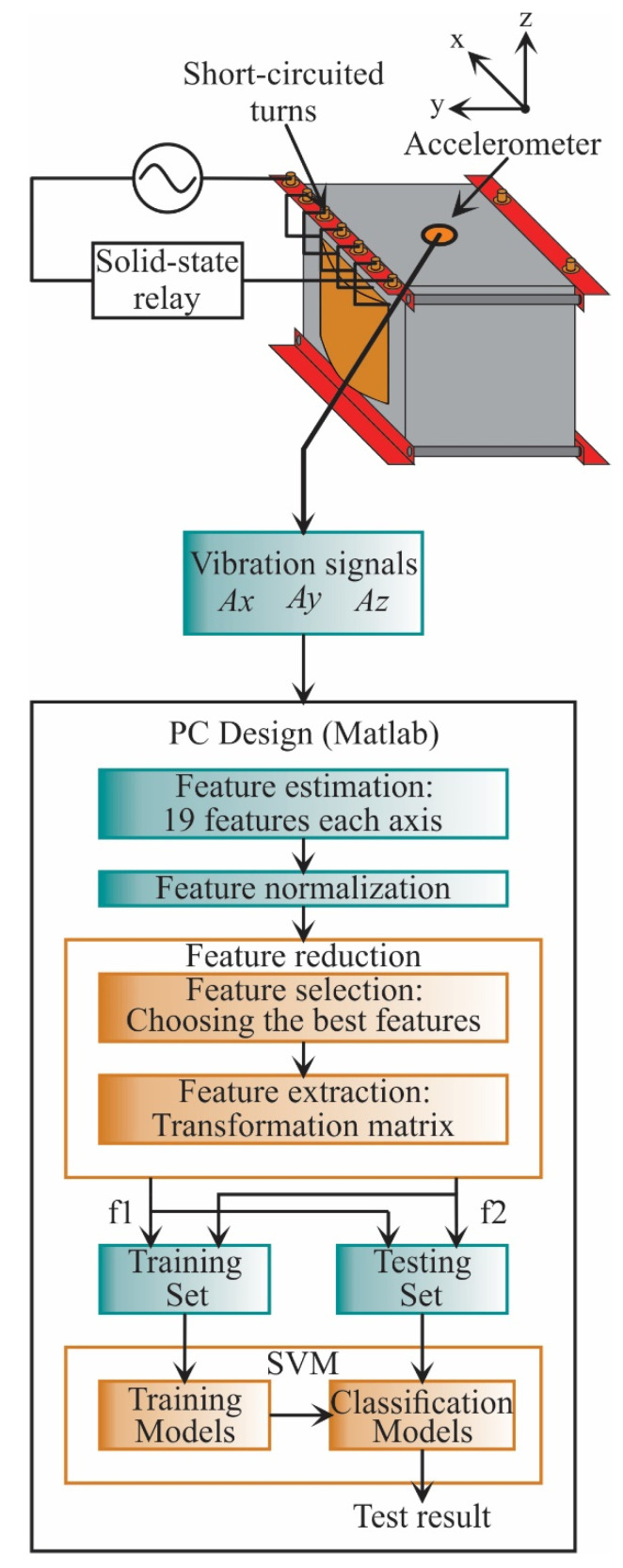
Flowchart for the proposed methodology during the design stage.

In the design stage (see Figure 4), the vibration signals acquired are from the three axes ($A_{x}$, $A_{y}$, $A_{z}$). These signals are acquired from a single-phase transformed without load during its energization; therefore, the vibration signals include transient state and steady-state. The transformer can emulate diverse conditions of SCTs: 0, 5, 10, 15, 20, 25, 30, and 35, where 0 SCT is the healthy condition. The energization is carried out through a solid-state relay.

In the design stage, the 19 STFs are calculated for each axis to find properties that can describe the behavior of the vibration signal. The analyzed STFs are specified in Table 1, Section 2.2. When the STFs are calculated, they are also normalized to obtain a suitable measure to compare the extracted features. Then, the feature reduction, which consists of feature selection and feature extraction, is carried out. In the feature selection, a subset of features that maximize the distance between classes and minimize the distance between data points are selected. Meanwhile, the feature extraction uses a transformation matrix to decrease the *n*-dimensional space to a 2-dimensional space: feature 1 (f1) and feature 2 (f2). Once the data have been obtained, they are separated into two groups: a training set and a testing set. The training set is used to construct the SVM models, i.e., to train the SVM, and the testing set is used to evaluate the SVM models and validate the classification results.

### 3.2. Implementation Stage

**Figure 5:**
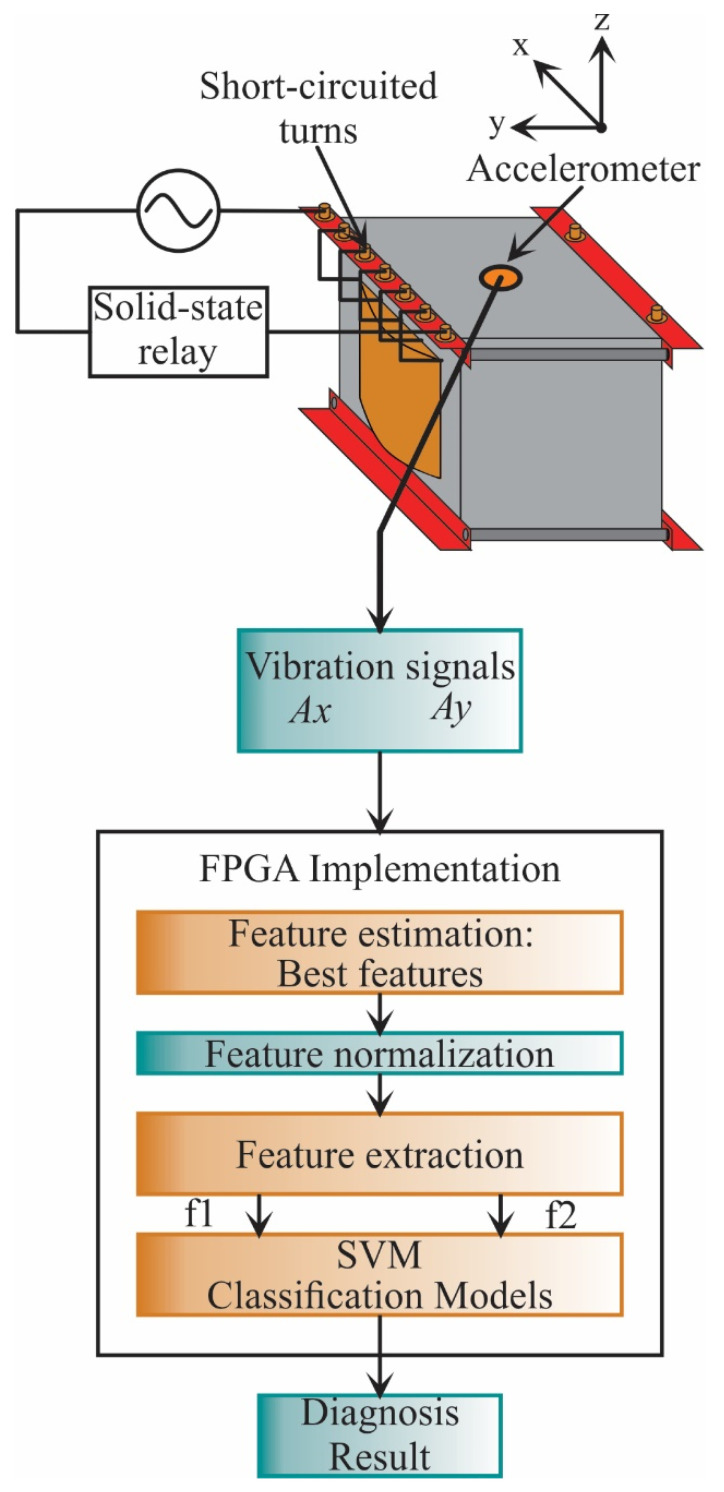
Flowchart for the proposed methodology during the implementation stage.

The implementation stage (see Figure 5) utilizes the best features found in the design stage. These features are only acquired from two axes ($A_{x}$, $A_{y}$); in the design stage, it was observed that the estimated features from the z-axis do not present relevant information and, therefore, are discarded. Then, the features are calculated as the first step, and then they are normalized. Next, the data is reduced to a 2-dimensional space using the transformation matrix calculated in the design stage. Finally, the classification unit based on SVM classifiers, which was constructed and validated in the design stage, diagnoses the transformer condition. It is worth noting that the implementation stage can be carried out in a personal computer, e.g., using Matlab software as was used in the design stage; yet, in this work, to provide a system-on-a-chip-solution for future equipment development, the FPGA implementation is developed. In this regard, the information used to normalize the features, the transformation matrix, and the values that describe the SVM models are stored in a read-only memory (ROM), included in the FPGA. The SVM-based classification stage determines the SCTs condition, showing values of 0, 1, 2, 3, 4, 5, 6, and 7, where class 0 represents the healthy condition, class 1 represents 5 SCTs, and so on.

## 4. Experiments and Results

This section shows the experimental setup, PC results, and FPGA results.

### 4.1. Experimental Setup

**Figure 6:**
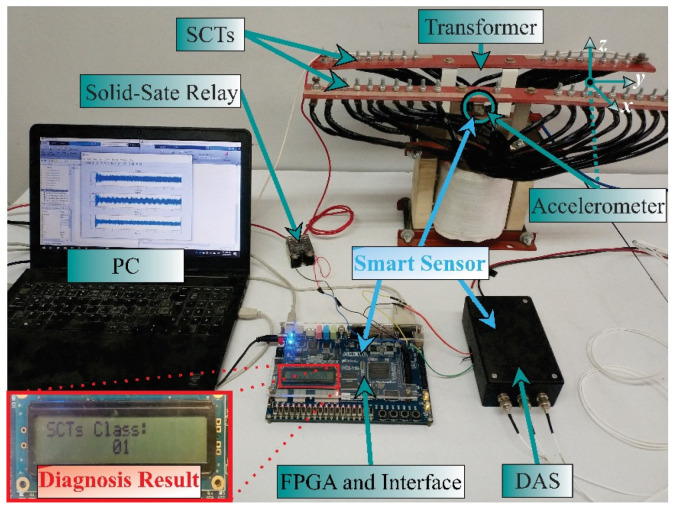
Experimental setup.

**Figure 7:**
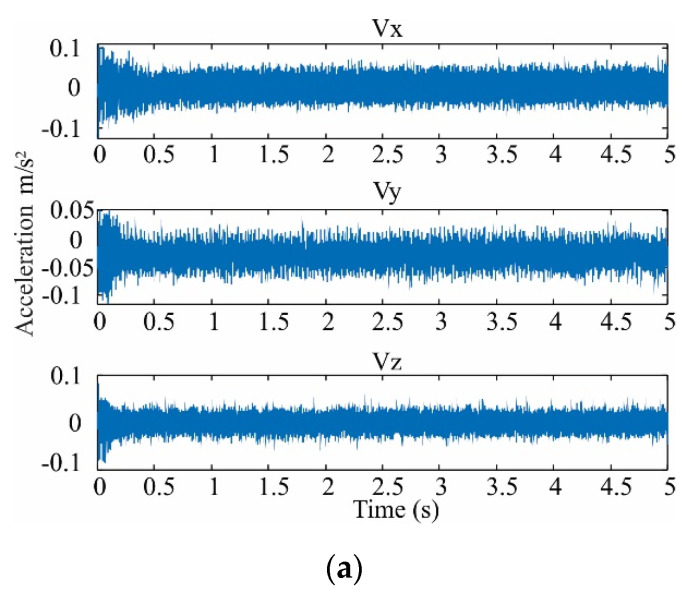

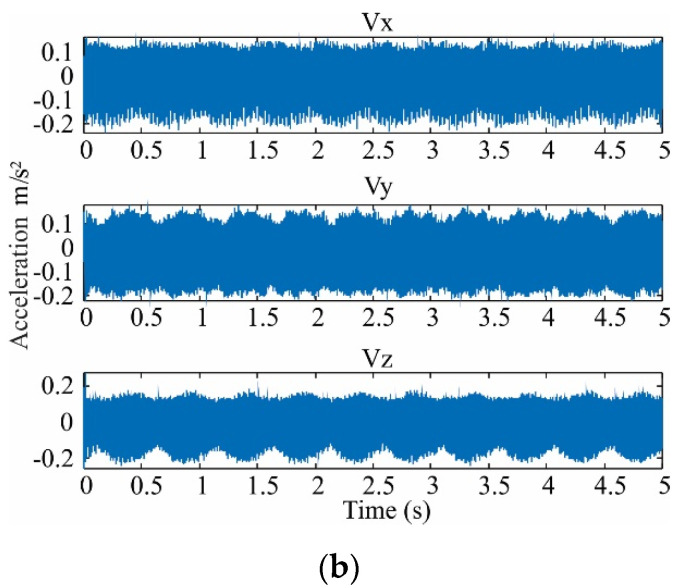
Vibration signals acquired in three directions for (**a**) healthy condition and (**b**) damage of 35 SCTs.

The experimental setup to test and validate the proposal is shown in Figure 6. A single-phase transformer of 1.5 kVA operated to 120 V is used. The transformer has 135 turns in its primary winding, which was modified to emulate several SCTs faults. These conditions are 0, 5, 10, 15, 20, 25, 30, 35 SCTs, where 0 SCT is the healthy condition. The vibration signals are measured from the transformer employing a triaxial accelerometer model 8395A from KISTLER, which can measure ±10 g with a resolution of 400 mV/g over a bandwidth of 1000 Hz. The accelerometer is located on the clamping frame of the transformer, where it can receive the vibrations from the core and the windings in a symmetrical way. The accelerometer data for the design stage (see Figure 4) are acquired employing a data acquisition system (DAS) based on the National Instruments NI-USB6211 board, which has a 16-bit analog-to-digital converter configured with a sampling frequency of 6000 samples/s. A solid-state relay model SAP4050D is employed to energize the transformer and start the test. The acquisition time is 5 s, enough to include the transient state and steady-state for each test during the transformer energization. Figure 7 shows an example of vibration signals acquired from the transformer in a healthy condition and a damage condition, respectively. At the end of each test, the transformer is de-energized with the activation of the autotransformer to eliminate the remnant flux. For each condition, 20 tests were performed. The resulting data are processed on a PC by using Matlab software. The PC has Windows 10 with a processor i7-4510U at 2.6 GHz and 8 GB of RAM. On the other hand, during the implementation stage, the complete processing is carried out into the FPGA by considering that the proposed methodology was previously designed. The used FPGA is a Cyclone IV EP4CE115F29C7 into an Altera DE2-115 development board. In Figure 6, a zoomed area of the liquid crystal display from the development board to show the SCTs class during a 5 SCTs fault identification is shown. It is worth noting that the proposed system can be considered as a smart sensor since it contains an accelerometer as a primary sensor, a DAS, and processing capabilities with communication protocols. However, the development of a smart sensor will need both optimization and design stages to convert the proposed system into a portable smart sensor-based monitoring system.

### 4.2. Design Results

#### 4.2.1. Feature Estimation and Normalization

**Figure 8:**
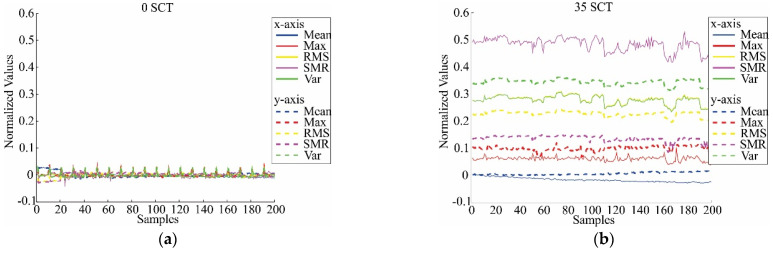

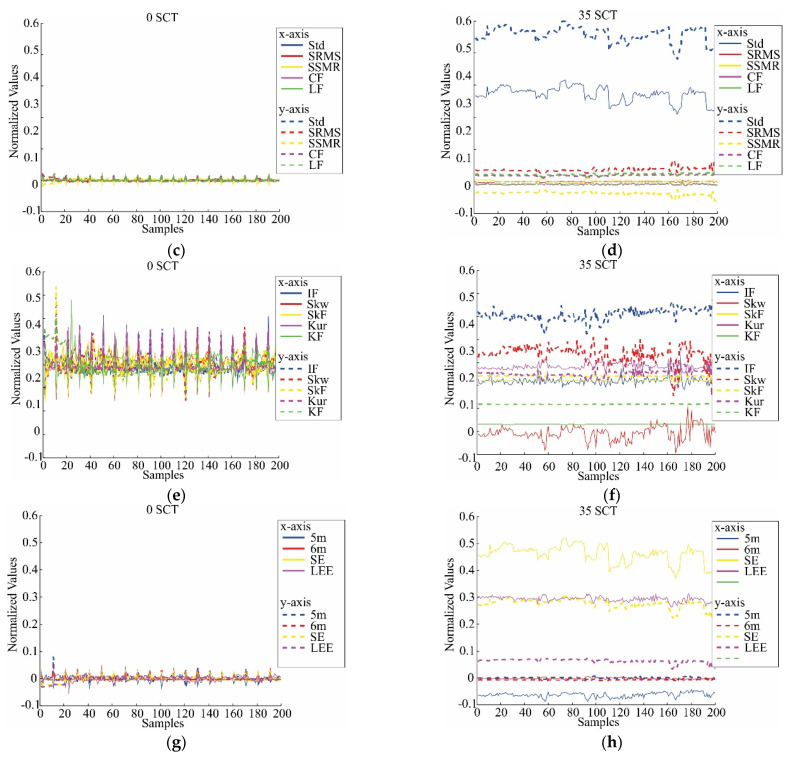
Estimated normalized features in healthy condition and 35 SCTs fault condition: (**a**) Mean, maximum value (Max), RMS, SRM, and variance (Var) for 0 SCT; (**b**) Mean, maximum value (Max), RMS, SRM, and variance (Var) for 35 SCT; (**c**) Standard deviation (Std), shape factor (RMS) (SRMS), shape factor (SRM) (SSRM), crest factor (CF), and latitude factor (LF) for 0 SCT; (**d**) Standard deviation (Std), shape factor (RMS) (SRMS), shape factor (SRM) (SSRM), crest factor (CF), and latitude factor (LF) for 35 SCT; (**e**) Impulse factor (IF), skewness (Skw), skewness factor (SkF), kurtosis (Kur), and kurtosis factor (KF) for 0 SCT; (**f**) Impulse factor (IF), skewness (Skw), skewness factor (SkF), kurtosis (Kur), and kurtosis factor (KF) for 35 SCT; (**g**) 5th Normalized moment (5m), 6th normalized moment (6m), Shannon entropy (SE), and log energy entropy (LEE) for 0 SCT; (**h**) 5th Normalized moment (5m), 6th normalized moment (6m), Shannon entropy (SE), and log energy entropy (LEE) for 35 SCT.

The feature estimation is carried out by analyzing the vibration signals in time windows of 0.5 s. This time window length is selected because the transient state lasts approximately half-second in the tests; thus, the features will be able to describe its behavior. As each signal lasts 5 s, 10 time windows are obtained from each vibration signal. For each time window, 19 STFs are computed. Therefore, a data matrix of 1600×57 is created, i.e., 20tests⋅10time_windows⋅8SCT_conditions×19STFs⋅3axes. In a subsequent step, it was observed that the estimated features from the z-axis do not present relevant information; therefore, they are discarded to avoid computational burden during the design stage, resulting in a data matrix of 1600×38. To have the same reference, all the features are normalized by using [26]:

(36) $$X=\frac{x-\mu_{0}}{\sigma_{0}},$$

where **x** represents each column of the matrix data, $\mu_{0}$ and $\sigma_{0}$ are the mean and standard deviation of **X** for the healthy condition only to take this condition as the reference. As an example of these results, the normalized features; i.e., values between −1 and 1 without units, for 0 SCTs and 35 SCTs conditions are shown in Figure 8; as can be observed, there is a clear difference between the classes, indicating the possibility of establishing an automatic classifier. The number of samples is 200 for each SCTs condition since there are 20 different tests and each test allows 10 time windows of 0.5 s.

#### 4.2.2. Feature Selection

**Figure 9:**
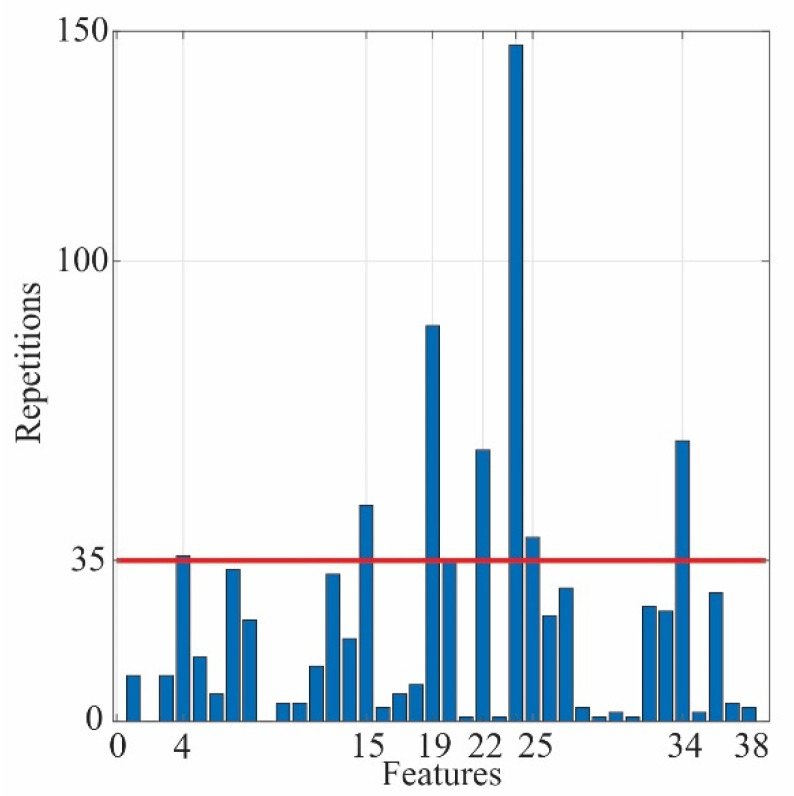
Histogram for the best features.

A significant number of features do not mean that all of them provide representative information to describe the behavior of the system or, in this case, the SCTs conditions, as this information can be redundant or irrelevant. In this regard, feature selection aims to filter these features to obtain a more informative subset and, at the same time, minimize their redundancy. This process is carried out by computing the Fisher score, which is a relative measure in terms of distance between the data points from diverse classes. A large Fisher score value represents a considerable distance between features; hence, these features are more relevant. However, the method must evaluate a total of $2^{m}-1$ subsets, where *m* represents the total number of features in the data set. In this case, the number of features is 38, i.e., 19 for the x-axis and 19 for the y-axis. The evaluation of all these subsets is computationally infeasible; then, a heuristic method can be proposed to find a proper subset of features without an expensive computational burden [38]. A three-feature combination subset is generated in the first place, 8436 different subsets are created. Subsequently, these subsets are evaluated through the Fisher score, and the best 10 combinations are selected, i.e., the subsets with the 10 highest Fisher score values. This process must be replicated for all combinations of conditions to find the maximum separability between all classes. In this way, having eight conditions, the number of combinations is 28, implying 236,208 Fisher score calculations, which is negligible compared with $2^{m}-1$. A histogram that shows the features that most maximize the separation between classes can be obtained (see Figure 9). There, the features with the biggest numbers of appearances and above a threshold value are adopted to be part of the new subset; in this work, the threshold value of 35 is selected by trial and error, looking for the best classification results, as will be shown in Section 4.2.4. In this regard, the new subset of features is selected. It contains the features: 4, 15, 19, 22, 24, 25, and 34, as shown in Figure 9. These features correspond to SRM, kurtosis factor, and LEE for x-axis and RMS, standard deviation, variance, and kurtosis factor for the y-axis.

#### 4.2.3. Feature Extraction

**Figure 10:**
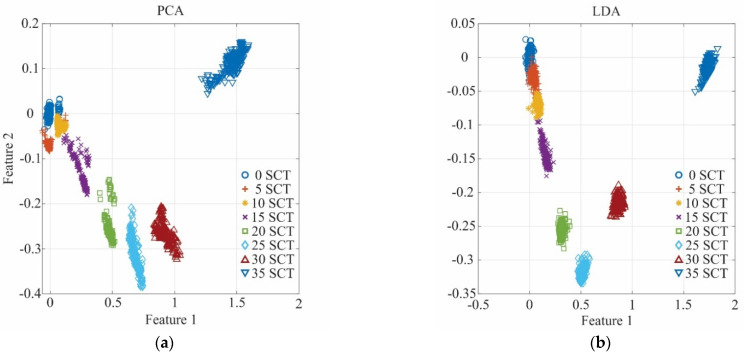
Performance comparison between (**a**) PCA transformation and (**b**) LDA transformation.

In this process, a new feature subset of 2-dimensional transformation from the original subset is obtained. As abovementioned, LDA and PCA can offer a different transformation for the data set. In Figure 10, the results for the transformations using both techniques are shown. Figure 10a shows the transformation carried out by PCA, whereas Figure 10b shows the LDA transformation. Although good results are obtained in both transformations, a better grouping in terms of area is observed for LDA; therefore, it is selected as the transformation step. 

#### 4.2.4. Classification Results

An SVM was trained to carry out the classification task by using MATLAB software. As σ and *C* parameters of the SVM have a significant influence on the classification accuracy, an exhaustive analysis in a trial-and-error process was carried out, resulting in values of 0.25 and 50, respectively. The training and validation steps were carried out using a *k*-fold validation with a *k* value of five. To compare the performance of the SVM, an ANN was used. The ANN parameters were also chosen in a trial-and-error process, resulting in an ANN with seven inputs (i.e., seven features), nine neurons in the hidden layer, and eight neurons (i.e., eight SCTs conditions) in the output layer. The activation functions are log-sigmoid for the input and hidden layers and linear for the output layer. The obtained results show that the SVM has a better performance than the ANN when the same number of features is used. The results of both classifiers are presented in Table 2.

As can be seen in Table 2, the SVM has better performance than ANN in each subset of features chosen. The number of features in each subset is defined by the threshold value obtained in the feature selection (see Figure 9). For both classifiers, the best results are when all the features are used, i.e., a threshold of 0. To keep a balance between the number of features and the classification performance, the threshold of 35, i.e., 7 features, is chosen. This subset has better performance than the subset of 10 features and only decreases one percent compared to the subset of 38 features, which implies a noticeable reduction of the computational burden.

**Figure 11:**
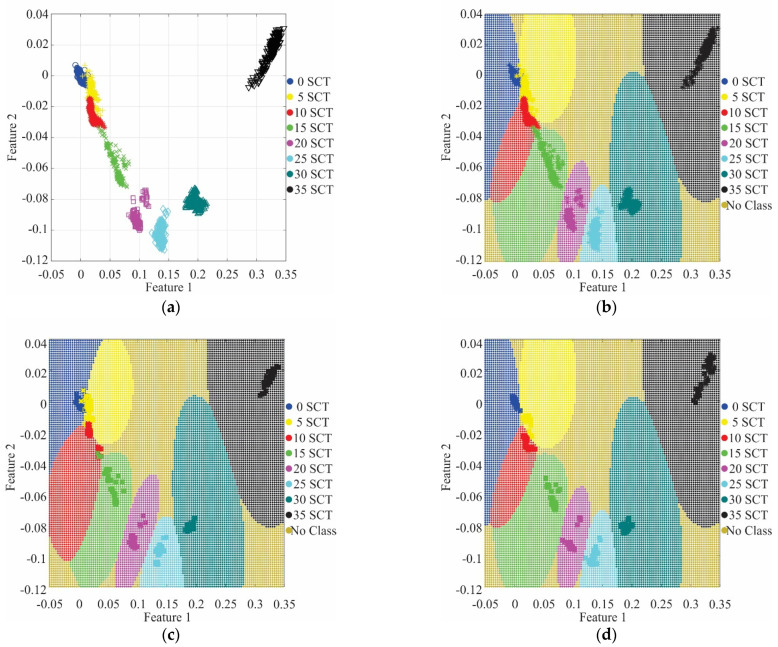
SVM classification: (**a**) dataset using 7 features; (**b**) projection of decision regions and his training data from a five-fold; (**c**) projection of decision regions and validation data from a *k* = 4; (**d**) projection of decision regions and validation data from *k* = 3.

Figure 11 shows the clusters and their decision regions for the SVM results using a *k*-fold validation. In particular, Figure 11a shows the clusters produced for a threshold of 35, i.e., seven features, and the LDA transformation for these features. It can be noted that for the initial conditions: healthy (blue), 5 SCTs (yellow), 10 SCTs (red), and some data points of 15 SCTs (green), the clusters are close to each other, even some points are mixed; yet, it is possible to differentiate them and define the borders between their decision regions as shown in Figure 11b. The remaining clusters (20, 25, 30, and 35 SCTs) do not present any complications. Figure 11c,d show the results for different *k* values in the five-fold validation. Figure 11c shows the results for *k* = 4, where some points misclassified can be observed, principally data points from the 5 SCTs and 10 SCTs conditions; yet, the accuracy percentage keeps above 90%. Figure 11d shows the results for *k* = 3, in which it can be seen that most of the validation data points were classified adequately. Table 3 shows the confusion matrix using five-fold validation when *k* = 3, where it can be observed that only one data is misclassified, obtaining 99.7 % of effectiveness. It is worth noting that for any condition that has not to be contemplated in the training step, the resulting class will follow the regions shown in Figure 11d, i.e., it can be No Class or be one of the two adjacent classes (e.g., for a 7 SCT condition, the resulting class can be either 5 SCT condition or 10 SCT condition according to the decision region). For a better resolution and accuracy in the classification of the fault severity, further research for interpolated and extrapolated conditions has to be carried out.

### 4.3. FPGA Implementation

Once the methodology has been developed and validated, the FPGA-based hardware solution is also implemented for future equipment development such as smart sensors [43,64], flexible systems that include software [65,66], different sensors with real implementations [67], among others.

**Figure 12:**
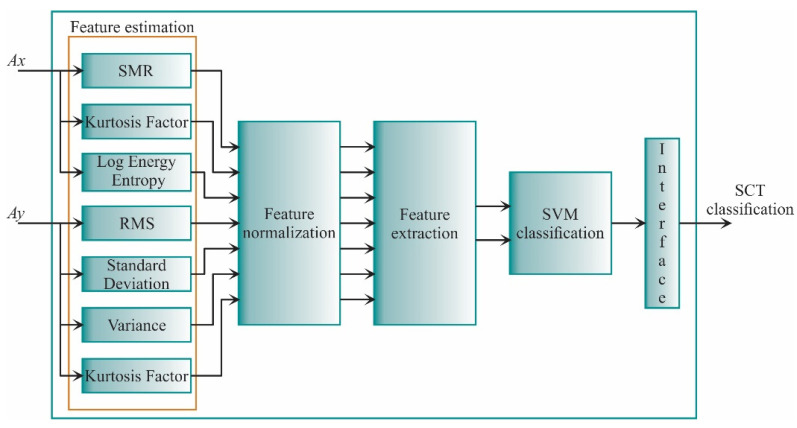
The principal architecture of the FPGA-based processor.

Figure 12 shows the principal architecture for the proposed FPGA processor. It follows the structure presented in Figure 5. In general, the FPGA-based processor consists of four main steps. The first step is the feature estimation in which the STFs are computed for the vibration signals of the x-axis and y-axis. The second and third stages are feature normalization and feature extraction. Finally, the SVM classification stage classifies the new sample into a particular SCTs condition. All these stages are described in detail in the next subsections.

#### 4.3.1. Statistical Time Features Estimation

**Figure 13:**
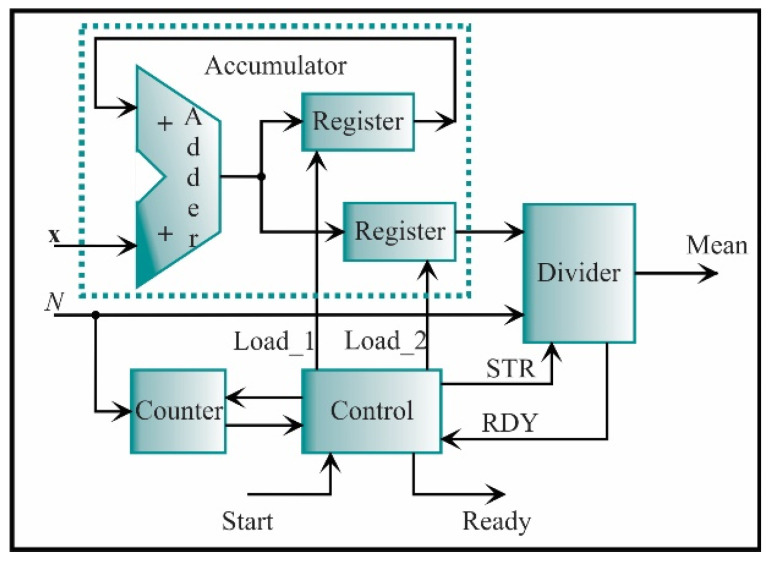
The architecture of the processing core for computing the mean.

For this stage, seven features have to be estimated, i.e., three for the x-axis and four for the y-axis. Nevertheless, one of these features appears in both axes, i.e., the kurtosis factor. Therefore, six processing cores will be only constructed. Figure 13 shows the core for the mean, which is given by Equation (1). This core is based on accumulator structure. The function of this structure is to compute successive sums using one adder and two registers. The adder output is connected to the inputs of the registers; the output of one of them is connected in feedback to the adder to contain the cumulative value of N−1 successive sums of the input signal, **x**, where *N* is the number of samples; the word length of each **x** data is *e* = 2 and *f* = 16. The other register will contain the total cumulative value until the count concludes. Each register has a load signal to maintain the respective values. From now on, the accumulator structure will be represented as a single block and will not have the load signals. The accumulator output goes to a divider to be divided by the number of samples *N* and obtain the mean. The divider is a digital structure based on a successive approximation register (SAR). Taking the division as *A/B = Y*, the SAR successively approximates the quotient *Y* by comparing the product of the quotient and the divisor (Y×B) with the dividend *A* until the product is equal or very close to the dividend. Also, the divider includes the signal to initiate the process (STR) and the signal that indicates the process ending (RDY). For clarity, the load, start, and ready signals from registers, accumulators, and dividers will also be omitted in the next diagrams. For completing the structure, a counter is used to count from zero to *N*-1 when the control unit indicates it. The control unit synchronizes the start and the end of the complete process. 

**Figure 14:**
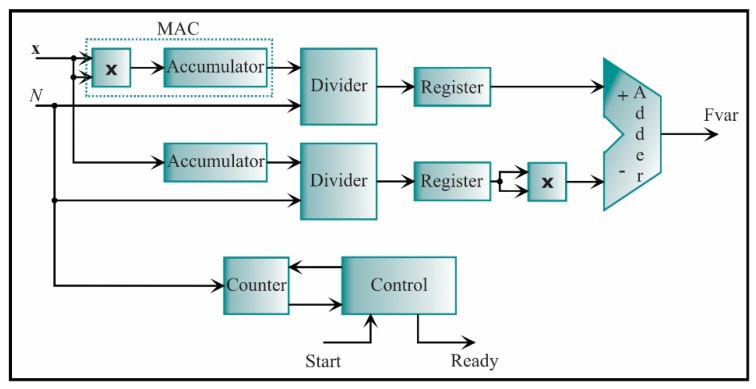
The architecture of the processing core for computing the variance.

The variance processing core is shown in Figure 14. Rearranging Equation (7), the variance can be computed as

(37) $$F_{\mathrm{var}}=\frac{1}{N}\sum_{i=1}^{N}x_{i}^{2}-{\left[\frac{1}{N}\sum_{i=1}^{N}x_{i}\right]}^{2}$$

With this equation, the variance processing core is constructed. This core is based on the digital structure called multiplier-accumulator (MAC). It computes a successive sum of products. Also, an accumulator is employed to cumulate the input values, *x*. The output signals of the accumulator are connected to a divider and, next to, a register for storage. Then, a subtraction between the first term and the second term of Equation (37) is carried out, where the second term is squared.

**Figure 15:**
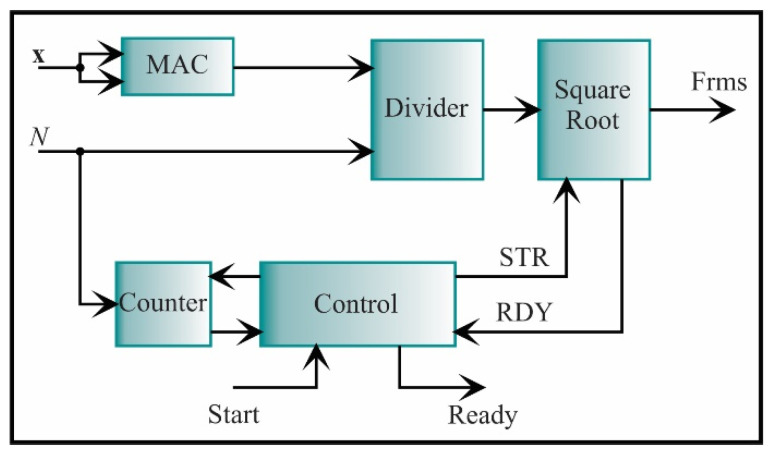
The architecture of the processing core for computing the RMS.

In Figure 15, the RMS architecture, which is described by Equation (5), is shown. It consists of a MAC, a divider, and a square root unit. The square root is similar to the divider, i.e., it is based on a SAR, but they differ in the comparison. For the square root, the root square value is compared with the squared radicand until this value is equal or very close to the root value. The signals to control the square root are start and ready. These signals start and finish the process. For clarity, they will be omitted in the next diagrams. The output of the square root block is the RMS value.

**Figure 16:**
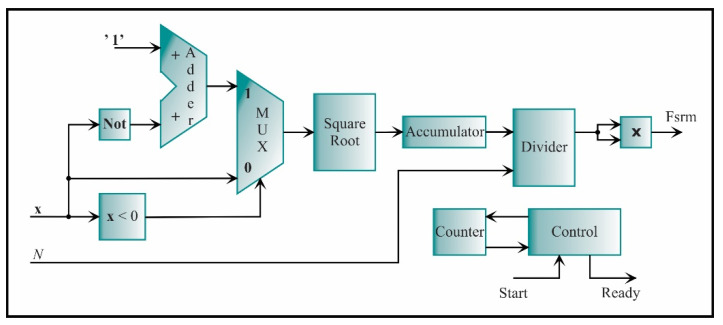
The architecture of the processing core for computing the SRM.

The SRM core is described by Equation (6) and shown in Figure 16. In the first place, the absolute value is computed. To always obtain a positive number, it is necessary to recognize the sign of the number; this is possible by comparing the most significant bit; if the most significant bit is one, the number is negative; otherwise, it is positive. If the number is negative, then the two’s complement is applied to obtain a positive number. The next step is to compute the square root of this number and accumulate it. When all the values were accumulated, the output value is divided by the number of data, *N*. Finally, the result of the division is squared, giving the SRM value. 

**Figure 17:**
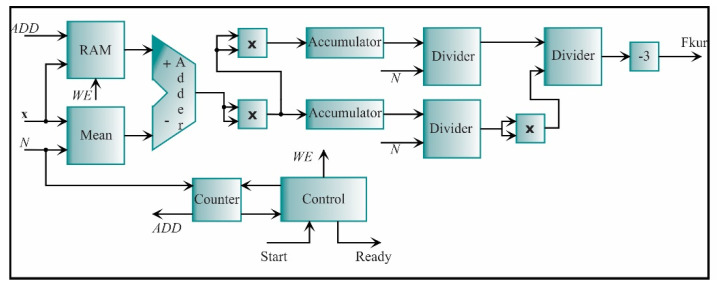
The architecture of the processing core for computing the kurtosis.

The next processing core is the kurtosis factor. It is described by Equation (17). However, it is necessary to first compute the kurtosis index. In this regard, Figure 17 shows the architecture of the kurtosis according to Equation (16) by using cores previously described, such as adders, multipliers, dividers, and accumulators. To compute the kurtosis, it is necessary to execute a subtraction between the input data and the mean of all the input data; therefore, the mean is first computed according to Equation (3) using the architecture presented in Figure 13. While the mean is computed, the input data is stored in a random access memory (RAM); additionally, the RAM has the input *ADD* to indicate the direction in which the input data will be stored when the input WE enable the option of writing it. Then, the subtraction can be computed. The result goes to a serial multiplier, and the product goes to two accumulators. One accumulator accumulates the subtraction elevated to the fourth power, and the other one accumulates the square subtraction. The output of each accumulator is connected to a divider, dividing the accumulated values by *N*. Both results are connected to another divider, where the dividend goes direct; meanwhile, the divisor passes by a multiplier to be squared. Finally, as the kurtosis value of the normal distribution is three, it is necessary to subtract this value from the resulting value to obtain the kurtosis value of a zero-value normal distribution. 

**Figure 18:**
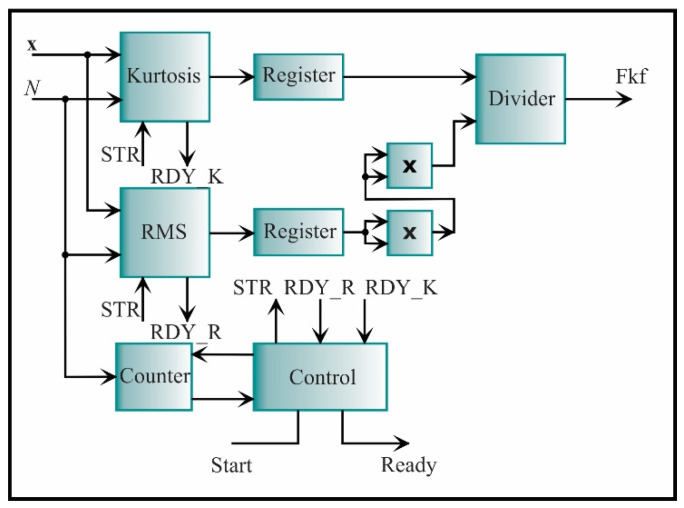
The architecture of the processing core for computing the kurtosis factor.

The architecture of the kurtosis factor is presented in Figure 18. It consists of two essential cores: the kurtosis and RMS, which were previously described. The signal to initiate both processes is STR and the signals that indicate the end of the process is RDY_K for the kurtosis and RDY_R for the RMS. The value of each core is stored in a register. The RMS value is elevated to the fourth power, becoming the divisor of the kurtosis value. The resulting value is the kurtosis factor value.

**Figure 19:**
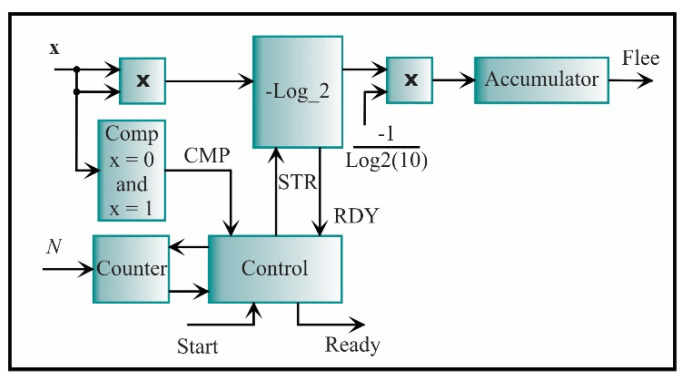
The architecture of the processing core for computing the log energy entropy.

The latter feature is the LEE, which is described by Equation (21). The proposed core for its computing is shown in Figure 19. In general, the LEE corresponds to the successive accumulation of logarithms of a squared signal. To do so, a multiplier for obtaining the squared signal is used, while the logarithm is estimated using Mitchell’s algorithm [68] and logarithm properties. The -Log_2 block initiates the process through the signal STR and the signal that indicates the end of the process is RDY. As the input signal cannot be zero or one, a comparator (Comp) is previously used. Finally, the control unit synchronizes the complete process taking the value from the comparator (CMP) and starting the -Log_2 block, giving through the accumulator the LEE value.

#### 4.3.2. Feature Normalization and Feature Extraction

**Figure 20:**
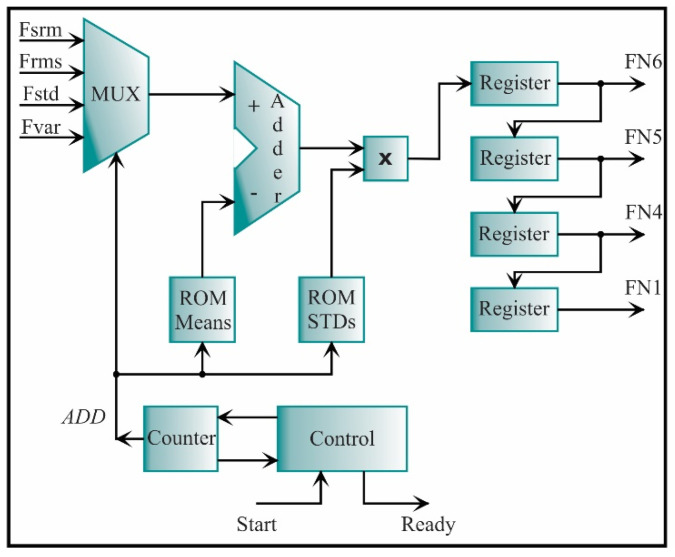
Architecture for computing the feature normalization of 4 features.

The generic architecture for feature normalization is depicted in Figure 20. In general, the architecture is constituted by a multiplexor, an adder, a multiplier, two read only memories (ROMs), and one register for each feature. The mean and standard deviation values, which were previously computed during the design stage, are stored in the ROM Means and ROM STDs, respectively. In Equation (36), the subtraction is divided by the standard deviation; yet, in this work, a multiplier is used to obtain the product among the subtraction and the inverse of the standard deviation. The multiplexor allows selecting the feature that will be normalized. The multiplexor reduces the resources since one adder, and one multiplier are only used for all the features. The normalized features (FN1, FN4, FN5, and FN6) are cascaded through a set of registers. 

**Figure 21:**
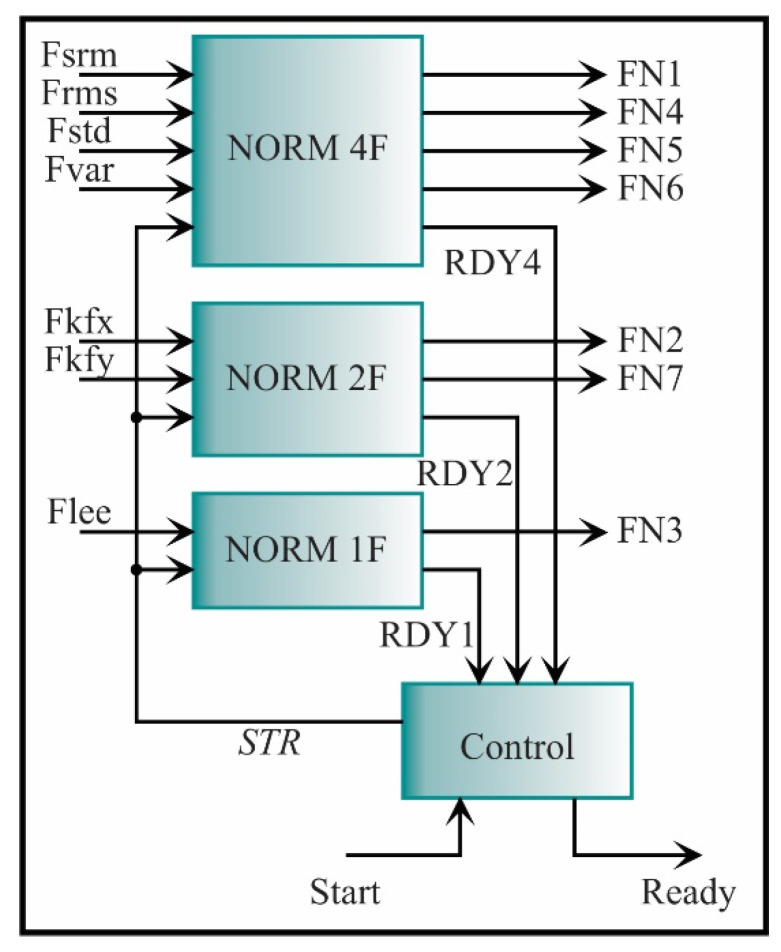
Architecture for computing the feature normalization.

Because the seven estimated features have different length words, the architecture presented in Figure 20 has to be modified according to the number of features that have the same word length; then, the feature normalization can be computed for all features (FN1, FN2, FN3, FN4, FN5, FN6, FN7). For instance, the SRM from the x-axis, RMS, STD, and variance from the y-axis has the same length word, i.e., *e* = 2 and f = 16; therefore, the architecture for these features (see Figure 19 and Figure 20) is considered as a single block. On the other hand, the kurtosis factor is estimated for both axes (Fkfx, Fkfy) with a word length of *e* = 24 and *f* = 16; therefore, two inputs, two registers, and the ROMs with two respective values are needed. For the LEE feature estimated from the x-axis, a word length of *e* = 16 and *f* = 16 is needed; therefore, one input, one register, and one value in each ROM are needed. In summary, the feature normalization architecture for all the features is depicted in Figure 21. The seven estimated features are the inputs. The NORM 4F is the feature normalization from Figure 20, while the NORM 2F and NORM 1f are the same architectures but they are modified for different word lengths, i.e., *e* = 24, *f* = 16, and *e* = 16, *f* = 16, respectively. For the seven features, the output signals have the same word length, i.e., *e* = 2 and *f* = 16.

**Figure 22:**
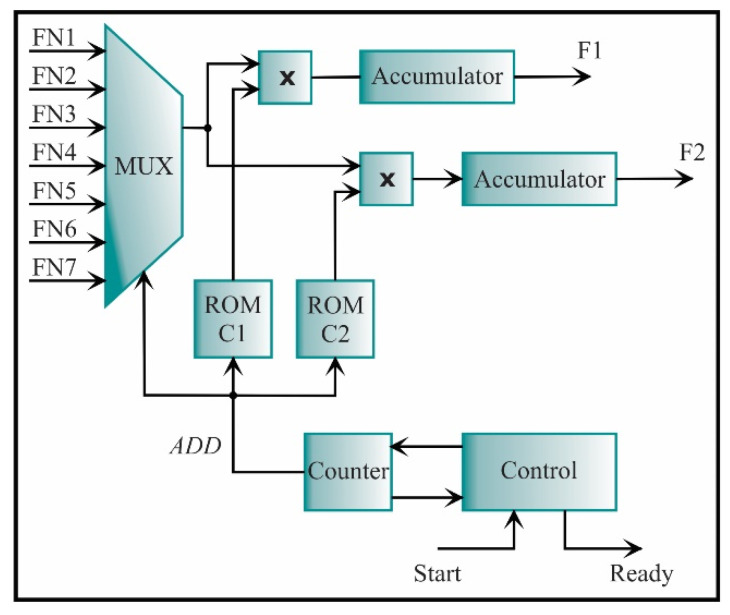
The architecture of the processing core for computing the feature extraction.

The feature extraction is carried out with the architecture depicted in Figure 22. It is obtained through a simple multiplication between the seven-feature vector and the transformation matrix, which is a 7×2 matrix built by the LDA method. In the ROMs C1 and C2, the transformation matrix is stored. The ROM C1 contains the values of the first column, and the ROM C2 contains the values of the second column. Multiplying and accumulating the features and the matrix elements, the new feature set in a lower dimension, i.e., dimension 2, is obtained to ease the classification step.

#### 4.3.3. SVM Classifier

**Figure 23:**
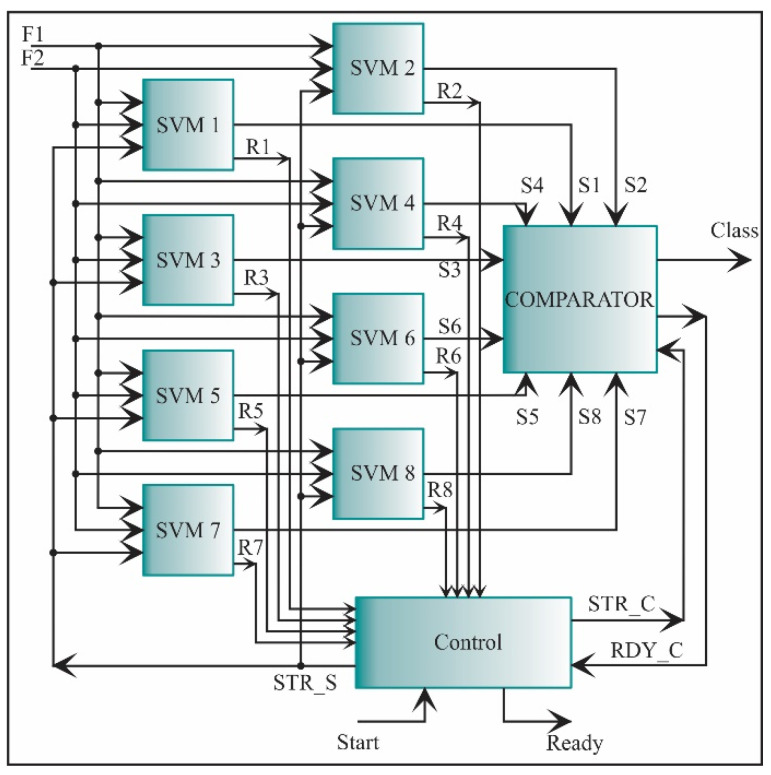
The architecture of the processing core for one-against-all SVM classification.

The multiple one-against-all structures are employed to classify a new data set. This structure allows separating one class from the others. The structure designed for this task is shown in Figure 23. It consists of eight SVM classifiers, i.e., one classifier for each condition. The inputs to each SVM core are the two features, i.e., the two features obtained in the feature extraction step. Then, each SVM core yields an output score (S1, S2, …, S8) according to Equation (35). If the score is positive, the new data set belongs to that class. However, there are regions in the feature space where no training data lie; in this case, different hyperplanes can yield positive or negative values. A comparator is required to evade this problem, and compare these score values, being the highest one the corresponding class for the new data set. The signal to initiate each SVM is STR_S, the signals that indicate the each SVM process ending are: R1, R2, …, R8, and the signals to initiate and finish the COMPARATOR are STR_C and RDY_C, respectively. 

**Figure 24:**
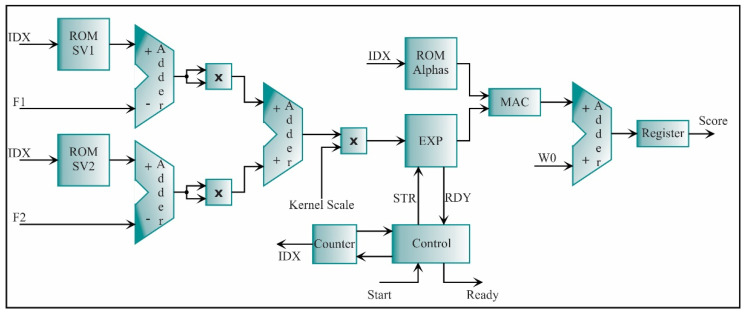
The architecture of the processing core for an SVM classifier.

The architecture of each SVM is constructed by following the Equation (33). The resulting processing core is shown in Figure 24. As abovementioned, the SVM core has three ROMs: two for the support vectors (ROM SV1 and ROM SV2) and one for the alpha values (ROM Alphas). The IDX signal indicates the address in each ROM. The block called EXP is an exponential function which is defined by the series:

(38) $$EXP(x)=\sum_{i=0}^{6}\frac{x^{i}}{i!}.$$

This series is used for its fast convergence and its easy implementation. The inverse of the dividends is used to avoid the divisions, into the multiplier since they are known in each iteration. This operation is implemented through a MAC. The blocks that complement the SVM core are adders, multipliers, an accumulator, and a register.

The kernel scale is taken as a constant value defined for each SVM core in the training step; thus, it can be utilized as a multiplication of a fractional number, avoiding the use of a divisor. The bias is represented by W0. Once the scores of the eight SVMs are obtained, the comparator of Figure 23 automatically determines the SCT condition.

#### 4.3.4. Results

In Table 4, the processing time of each processing core and their relative errors are shown. It can be noted that the processing time in clock cycles depends on: the number of data *N*, the width of each data (i.e., *e* and *f*, which are the integer and fractional parts of each datum, respectively), and the number of maximum support vectors Nsv. For this work, *N* = 3000 and Nsv = 192. As the SRM feature has the longest processing time by considering all the seven features, this time will reference the feature estimation stage. Therefore, the total number of cycles corresponds to the sum of the clock cycles of the SRM feature, feature normalization, feature extraction, and SVMs stages. The total number of cycles of the SVM stage is from the SVM with the biggest number of support vectors. 

Consequently, the total time using a 50 MHz clock is around 1.24 ms. The relative error is obtained by comparing the estimated values in Matlab using floating-point and the ones estimated by the processing cores using fixed-point. On the other hand, although the maximum relative error is two percent, this result can be considered accurate enough since the difference between values is of an order of magnitude of $10^{-3}$. 

Table 5 summarizes the FPGA resources used for each processing core. As can be observed, most of them occupy less than 2% of the total available resources, except for the number of multipliers. These results indicate that the proposed FPGA-based processor can be implemented either in the FPGA used in this work or many other commercially available FPGA devices with an equivalent number of multipliers but with much fewer elements, registers and, memory bits, which could reduce the implementation costs. 

## 5. Conclusions

In this paper, a new methodology and its implementation into an FPGA for assessing the transformer condition under different levels of SCT fault is presented. To test and validate the methodology and its FPGA implementation, vibration signals are acquired from a transformer that can emulate different SCT fault conditions. The obtained results show the potential of the methodology to carry out a transformer diagnosis. In this regard, the main findings are

- The methodology developed and implemented into the FPGA can diagnose eight severity levels of SCTs in a transformer by measuring the vibration signals from the top of the transformer core;
- The feature reduction allows obtaining the best set of features, selecting the features that present the most relevant information related to the transformer performance and then, reducing the dimensional space;
- The Fisher score implementation to select features allows reducing from an extensive number of features a set of only seven STFs, i.e., three for the x-axis: SRM, kurtosis factor, and LEE, and four for the y-axis: RMS, standard deviation, variance, and kurtosis factor;
- For reducing the dimensional space, the LDA method presents a more satisfactory performance than the PCA method, simplifying the classification process;
- The SVM classifier can classify among eight severities of SCT with an accuracy of 96.82%. The results also demonstrate that the SVM classifier performs better than an ANN under the same experimental setup;
- The processor core makes use of low FPGA resources, presents a maximum relative error of 2% if it is compared with its floating-point computation in Matlab software, and requires a small computing time (≈1.24 ms) to offer a diagnosis result;
- All these characteristics show the suitability of the FPGA technology for a future device development, e.g., a smart sensor since the accelerometer, the DAS, and the FPGA-based processor represents the basic elements that compose it;
- The proposed methodology and the individually developed cores could also be adaptable and calibrated to other applications such as assessment buildings, bridges, wind turbines, induction motors, and other types of equipment as demonstrated in the literature.

Future work will focus on employing the methodology to diagnose single-phase and three-phase transformers under different transformer operation conditions, e.g., with load unbalance and harmonic content in the power supply, among others. The impact of the fault location on the proposed methodology will be explored to assess its robustness and calibrate it if necessary, aiming at proposing a methodology that can detect, quantify, and locate a fault condition. Besides, different sets of features in time and frequency domains, fractals, and entropy-base indicators will be explored, as well as different optimization algorithms such as genetic algorithms [51] and boosting algorithms [69] will be used to improve the performance of the classifiers.

## Figures and Tables

**Table 1:** Statistical time features.

Feature	Equation		Feature	Equation	
Mean	$F_{mn}=\frac{1}{N}\sum_{i=1}^{N}x_{i}$	(3)	Impulse Factor	$F_{if}=\frac{F_{max}}{\frac{1}{N}\sum_{i=1}^{N}\left|x_{i}\right|}$	(13)
Maximum Value	$F_{\max}=\max(x)$	(4)	Skewness	$F_{skw}=\frac{\sum_{i=1}^{N}{\left(x_{i}-F_{mn}\right)}^{3}}{N\cdot F_{std}^{3}}$	(14)
RMS	$F_{rms}={\left(\frac{1}{N}\sum_{i=1}^{N}x_{i}^{2}\right)}^{1/2}$	(5)	Skewness Factor	$F_{skf}=\frac{F_{skw}}{F_{rms}^{3}}$	(15)
SRM	$F_{srm}={\left(\frac{1}{N}\sum_{i=1}^{N}{\left|x_{i}\right|}^{1/2}\right)}^{2}$	(6)	Kurtosis	$F_{kur}=\frac{\sum_{i=1}^{N}{\left(x_{i}-F_{mn}\right)}^{4}}{N\cdot F_{std}^{4}}$	(16)
Variance	$F_{\mathrm{var}}=\frac{1}{N}\sum_{i=1}^{N}{\left(x_{i}-F_{mn}\right)}^{2}$	(7)	Kurtosis Factor	$F_{kf}=\frac{F_{kur}}{F_{rms}^{4}}$	(17)
Standard Deviation	$F_{std}={\left(F_{\mathrm{var}}\right)}^{1/2}$	(8)	Normalized 5th central Moment	$F_{5m}=\frac{\sum_{i=1}^{N}{\left(x_{i}-F_{mn}\right)}^{5}}{N\cdot F_{std}^{5}}$	(18)
Shape Factor for RMS	$F_{srms}=\frac{F_{rms}}{\frac{1}{N}\sum_{i=1}^{N}\left|x_{i}\right|}$	(9)	Normalized 6th central Moment	$F_{6m}=\frac{\sum_{i=1}^{N}{\left(x_{i}-F_{mn}\right)}^{6}}{N\cdot F_{std}^{6}}$	(19)
Shape Factor for SRM	$F_{ssrm}=\frac{F_{srm}}{\frac{1}{N}\sum_{i=1}^{N}\left|x_{i}\right|}$	(10)	Shannon Entropy	$F_{se}=-\sum_{i=1}^{N}x_{i}^{2}\log\left(x_{i}^{2}\right)$	(20)
Crest Factor	$F_{cf}=\frac{F_{\max}}{F_{rms}}$	(11)	Log Energy Entropy	$F_{lee}=-\sum_{i=1}^{N}\log\left(x_{i}^{2}\right)$	(21)
Latitude Factor	$F_{lf}=\frac{F_{\max}}{F_{srm}}$	(12)			

**Table 2:** Accuracy percentage of SVM and ANN for different threshold values according to Figure 9.

Threshold	Features	SVM (%)	ANN (%)
0	38	97.76	95.29
30	10	96.76	93.98
35	7	96.82	94.97
40	5	96.64	94.33

**Table 3:** Confusion matrix of SCTs classification using five-fold validation and *k* = 3.

SCTs	0	5	10	15	20	25	30	35
0	40	0	0	0	0	0	0	0
5	0	40	1	0	0	0	0	0
10	0	0	39	0	0	0	0	0
15	0	0	0	40	0	0	0	0
20	0	0	0	0	40	0	0	0
25	0	0	0	0	0	40	0	0
30	0	0	0	0	0	0	40	0
35	0	0	0	0	0	0	0	40

**Table 4:** Time and relative error from each processing core.

Digital Structure	Word Length	Time (Clock Cycles)	Relative Error (%)
SVM	*e* = 7, *f* = 20	2+10Nsv	0.33
Feature Extraction	*e =* 2, *f =* 16	14	0.3
Feature Normalization	*e =* 2, *f =* 16	10	1.67
SRM	*e =* 2, *f =* 16	(N+1)(e+f+2)+1	2
Kurtosis Factor	*e =* 24, *f =* 16	4(e+f)+3N+35	0.05
Log Energy Entropy	*e =* 16, *f =* 16	N(f+2)+2	1.11
RMS	*e =* 2, *f =* 16	2(e+f)+N+5	0.004
Standard Deviation	*e =* 2, *f =* 16	2(e+f)+N+6	0.002
Variance	*e =* 2, *f =* 16	e+f+N+5	0.0002
**Total time with a** **50 MHz clock**		**61,967 clock cycles** **1,239,340 ns**	

**Table 5:** FPGA resources usage.

Digital Structure	Logic Elements (%)	Registers (%)	Multipliers 9-Bit (%)	Memory Bits (%)
SVM	6361 (6%)	1564 (1%)	256 (48%)	0 (0%)
Feature Extraction	178 (<1%)	101 (<1%)	4 (<1%)	73,728 (2%)
Feature Normalization	478 (<1%)	144 (<1%)	20 (4%)	0 (0%)
SRM	360 (<1%)	166 (<1%)	6 (1%)	0 (0%)
Kurtosis Factor	1818 (2%)	870 (<1%)	35 (7%)	0 (0%)
Log Energy Entropy	221 (<1%)	121 (<1%)	6 (1%)	0 (0%)
RMS	315 (<1%)	184 (<1%)	6 (1%)	0 (0%)
Standard Deviation	577 (<1%)	336 (<1%)	10 (2%)	0 (0%)
Variance	513 (<1%)	300 (<1%)	8 (2%)	0 (0%)
**Total** **Processor**	**12,639 (12%)**	**4656 (5%)**	**386 (73%)**	**73,728 (2%)**

## Data Availability

The data presented in this study are not publicly available due to privacy issues.
